# Evaluation of Body Position Association with Diuretic Response and Neurohormonal Activation in Patients with Acutely Decompensated Heart Failure

**DOI:** 10.3390/biomedicines14010209

**Published:** 2026-01-18

**Authors:** Mateusz Guzik, Rafał Tymków

**Affiliations:** 1 Institute of Heart Diseases, Faculty of Medicine, Wroclaw Medical University, 50-556 Wroclaw, Poland; 2 Department of Cardiology, Wroclaw Medical University Hospital, 50-556 Wroclaw, Poland

**Keywords:** congestion, diuretic response, neurohormonal activation, RAAS, natriuresis, acute heart failure, body position

## Abstract

**Background/Objectives:** Hemodynamic and neurohormonal factors, including renal perfusion and venous pressure, may affect diuretic response, which may be modulated by body position. This study aimed to assess whether supine versus upright positioning influences diuretic efficacy and neurohormonal activation during early decongestion in patients with AHF and reduced ejection fraction (HFrEF). **Methods:** This single-center, prospective, pilot randomized study enrolled 12 hospitalized patients with decompensated HFrEF receiving guideline-directed medical therapy. Participants were randomized (1:1) to remain in either the supine or upright/seated position during intravenous furosemide administration (1 mg/kg: half of the dose administered as a bolus, half as a 2-h infusion). Serial measurements of urine volume, electrolyte excretion, and neurohormonal biomarkers (renin, aldosterone, catecholamines) were performed at baseline, 2, and 6 h after diuretic administration. **Results:** No significant differences were found between supine and upright groups in total urine output, urine dilution, sodium excretion, or weight change after 6 h. There were no statistically significant differences in renin and aldosterone levels across subsequent timepoints; however, renin concentration tended to be higher in upright than in supine individuals. Interestingly, supine participants demonstrated greater urinary adrenaline concentration after furosemide administration, alone and after adjustment for urinary creatinine. **Conclusions:** No clinically meaningful differences were found between supine versus upright position patients with AHF, receiving neurohormonal blockade.

## 1. Introduction

The diuretic response is influenced by a range of factors, including renal perfusion and neurohormonal activity. Key regulatory systems such as the sympathetic nervous system, the renin–angiotensin–aldosterone system (RAAS), and vasopressin pathways modulate both vascular tone and intrinsic renal function, thereby affecting whole-body volume status [1,2,3,4,5,6,7,8].

In heart failure with reduced ejection fraction (HFrEF), pharmacologic agents targeting neurohormonal blockade constitute the cornerstone of evidence-based therapy. These interventions have been shown to improve outcomes; consequently, optimizing their modulatory effects on neurohormonal systems appears to be an important determinant of effective decongestion [9,10]. Adequate organ perfusion represents another critical determinant of diuretic efficacy, as urine formation depends not only on arterial pressure but also on venous pressure and vascular resistance [11]. Even in the presence of apparently satisfactory arterial blood pressure and relatively low systemic vascular resistance, elevated venous pressure can significantly reduce the renal perfusion gradient, leading to impaired renal function. In the setting of heart failure, diminished cardiac output results in reduced renal arterial flow, eliciting a compensatory increase in intrarenal vascular resistance aimed at preserving perfusion [12,13,14]. This phenomenon constitutes part of the so-called “stressed volume”—the intravascular volume responsible for generating pressure exceeding the mean circulatory filling pressure [12]. Beyond sympathetic activation, which induces fluid shifts and vasoconstriction, hydrostatic pressure also contributes to vascular dynamics depending on body position. Under physiological conditions, the hydrostatic component adds approximately 20 mmHg to the pressure at the level of the renal vein in the upright position relative to the right atrium. This may exert an unfavorable effect on renal vascular afterload and thereby reduce the renal perfusion gradient. Consequently, patients in the supine position are expected to be free from this adverse hydrostatic influence. This theory is supported by the results of a study conducted on an animal model (monkeys), which indicates that the head-up tilt from a supine position can alter the difference between the renal vein and the right atrium [15].

The clinical relevance of improving renal perfusion pressure in the context of diuretic therapy is underscored by ongoing investigations employing the Doraya catheter, designed to reduce renal venous pressure and thereby enhance renal perfusion [16]. Moreover, mechanistic observational studies conducted in diverse patient groups (hepatic cirrhosis, nephrotic syndrome, heart failure) indicated a tendency toward a better diuretic response, greater natriuresis, and lower plasma renin activity in patients who remained in a supine position compared with those who were standing during the study [17,18].

The introduction of drugs affecting the renin–angiotensin–aldosterone system provided some indications of mitigating the effect of body position on treatment response; however, the data have so far remained inconclusive [19,20]. Based on these pathophysiological considerations, we have designed a study to assess whether body position (supine vs. upright) influences the diuretic response in patients with acute heart failure.

## 2. Materials and Methods

The study was a single-center, prospective, pilot trial conducted from September to December 2024 at the Institute of Heart Diseases in Wroclaw, Poland. The study population consisted of patients hospitalized due to acute decompensation of chronic heart failure with reduced left ventricular ejection fraction (HFrEF) who required diuretic therapy, provided written informed consent to participate, and met all inclusion criteria without fulfilling any exclusion criteria. The study protocol was approved by the Bioethics Committee of the Wroclaw Medical University (approval no. 243/2024).

**Inclusion criteria:**

- Exacerbation of chronic heart failure symptoms;
- Stable, guideline-directed medical therapy (beta-blocker, ACEI/ARB/ARNI, MRA, SGLT2 inhibitor) for at least 3 months;
- Clinical signs of tissue congestion;
- Peripheral edema extending to at least the lower one-third of the shank.

**Exclusion criteria:**

- De novo acute heart failure;
- Pulmonary edema or cardiogenic shock;
- Severe tricuspid regurgitation;
- Chronic kidney disease with eGFR < 30 mL/min/1.73 m^2^.

### 2.1. Study Protocol

After enrollment, baseline demographic and clinical data were collected for all participants, including comorbidities, current pharmacotherapy, and physical signs of fluid overload. Each patient underwent urinary catheterization to accurately measure urine output.

Participants were randomized in a 1:1 ratio to either the supine or upright/seated position groups during diuretic treatment. Randomization was performed using a computer-generated random number sequence before study initiation. To account for circadian variations in neurohormonal parameters and to minimize inter-individual variability secondary to these rhythms, all study procedures were initiated at 8:00 AM. Furosemide was administered at 12:00 PM, and urine collection was completed at 6:00 PM.

All participants were instructed on the day before the study began to remain in the supine position for at least 4 h without leaving the bed prior to the start of the study. The first baseline samples were then obtained. Afterwards, half of the patients remained supine, while the other half changed to the upright position according to randomization. After this period, blood and urine samples were collected again for biochemical analyses. Subsequently, furosemide was administered at a dose of 1 mg/kg body weight—half as an intravenous bolus and half as a 2-h continuous infusion.

Hourly urine output was measured for 6 h following loop diuretic administration. Additional blood and urine samples were collected at 2 h and 6 h for further biochemical evaluation.

Throughout the study period, all patients adhered to a fluid restriction protocol and received hospital-prescribed meals in accordance with the standard heart failure diet.

Urine dilution was defined as urinary creatinine in baseline to urinary creatinine in subsequent timepoints. This parameter was designed to present the degree of water excretion changes in relation to baseline [21]. To adjust urine output for eGFR and to assess fractional sodium excretion (FeNa), baseline serum creatinine and sodium concentrations were used at all time points, assuming they remained constant.

### 2.2. Statistical Analysis

Quantitative data were expressed as mean ± standard error of mean (SEM). Qualitative data were presented as absolute numbers and percentages. Normality of distribution was assessed using the Shapiro–Wilk test, and homogeneity of variance was verified using Levene’s test. Statistical assessment of differences between groups was performed using Student’s *t*-test, with Welch’s correction applied in cases of unequal variances. For non-normally distributed variables, the Mann–Whitney U test was used.

All statistical analyses were performed using Statistica 13 (StatSoft, Inc., Tulsa, OK, USA). Graphs were generated using GraphPad Prism 9.5.0. The graphical abstract was created in BioRender (Biegus, J. (2026) https://BioRender.com/jw4tomi).

## 3. Results

### 3.1. Patients’ Characteristics

The study group included 12 patients, predominantly men (10 (83%)), with a mean age of 74 ± 4 years, with HFrEF. The mean ejection fraction was 28 ± 2%, NT-proBNP 15,162 ± 4490 pg/mL. The pre-diuretic urine output was 310 ± 83 mL (77 ± 21 mL/h), while cumulative 6-h diuresis after furosemide administration was 1855 ± 368 mL, (309 ± 61 mL/h). After adjustment for eGFR the mean hourly urine output in pre- and post- diuretic periods were: 1.29 ± 0.27 and 4.64 ± 0.69 mL/h per mL/min/1.72 m^2^ of eGFR, respectively. The mean furosemide dose administered during the study was 84 ± 5 mg. After 6 h of intensive diuretic treatment the weight change was −0.9 ± 0.4 kg, The comprehensive study group characteristic was presented in Table 1.

### 3.2. The Comparison of Urine Output Between Groups

**Figure 1:**
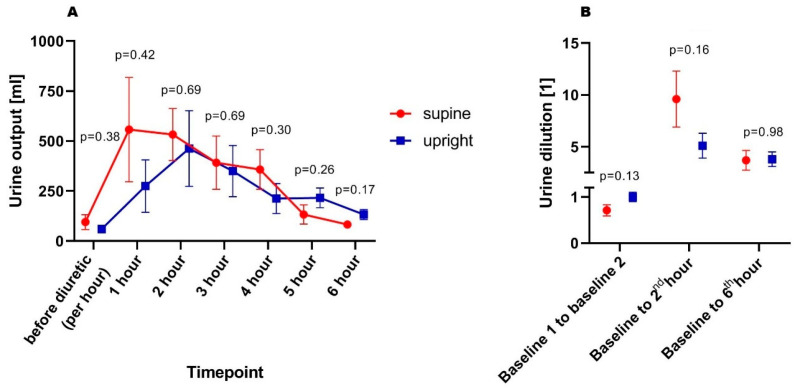
Urine output (**A**) and degree of urine dilution (**B**) in subsequent timepoints.

There were no differences between supine and upright patients regarding urine output in both pre-diuretic (94 ± 37 vs. 60 ± 19 mL/h; *p* = 0.37) and post-diuretic periods (343 ± 88 vs. 275 ± 91 mL/h; *p* = 0.58), respectively. Moreover, when adjusted for eGFR, there were no differences between supine and upright patients in both pre-diuretic (1.19 ± 0.43 vs. 1.40 ± 0.35 mL/h per mL/min/1.73 m^2^ of eGFR; *p* = 0.69) and post-furosemide periods (3.81 ± 0.62 vs. 5.52 ± 1.20 mL/h per mL/min/1.73 m^2^ of eGFR; *p* = 0.47). Naturally, it was not reflected in differences in weight change during 6 h of diuretic treatment between supine and upright individuals (−0.6 ± 0.4 vs. −1.1 ± 0.7 kg; *p* = 0.81). Moreover, when comparing cumulative urine output per 40 mg administered furosemide, the values of adjusted urine volume did not differ between each other (933 ± 192 vs. 835 ± 306 mL/40 mg furosemide; *p* = 0.69). However, patients randomized to the supine group presented a more dynamic diuretic response, reflected in a tendency to more diluted urine in 2nd hour after the diuretic dose. The urinary output and dilution trajectories were presented in Figure 1A,B.

### 3.3. Diuretic Response Biochemical Parameters Between Supine and Upright Patients

**Figure 2:**
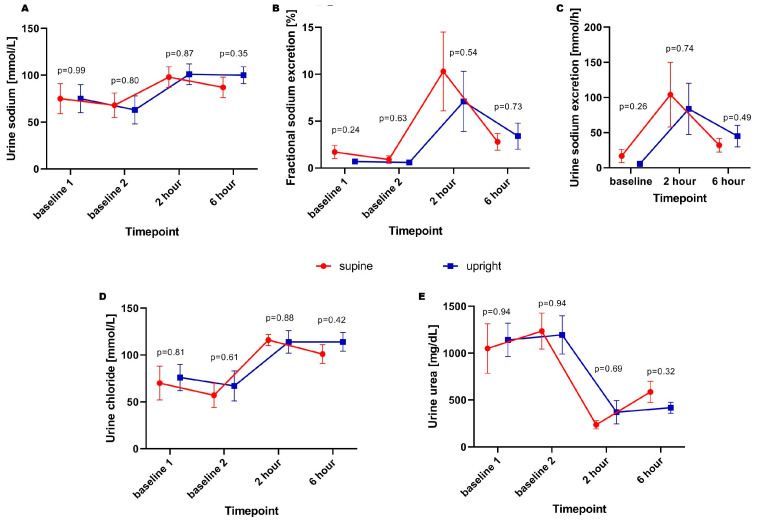
Spot urine sodium concentration (**A**), fractional (**B**) and total (**C**) excretion, urine chloride concentration (**D**), and urine urea concentration (**E**) in subsequent timepoints.

The trajectory of urine sodium concentration and excretion was similar in both supine and upright groups prior to loop diuretic dose and post-loop diuretic administration. Nevertheless, the slightly non-significant tendency for greater sodium fractional and total excretion prior to the loop-diuretic dose was observed (8.4 ± 4.6 vs. 2.8 ± 0.5 mmol/h; *p* = 0.26) in supine patients was observed. Moreover, there were no significant differences in fractional sodium excretion across all timepoints. The trajectories of urea and chloride excretion were parallel, with no significant differences across subsequent timepoints. The trajectories of the abovementioned parameters were presented on Figure 2A–E.

### 3.4. Neurohormonal Patterns in Supine and Upright Patients

**Figure 3:**
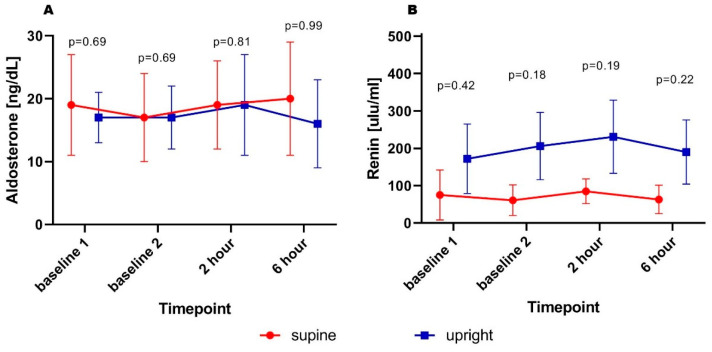
Serum aldosterone (**A**) and renin (**B**) concentration in subsequent timepoints.

**Figure 4:**
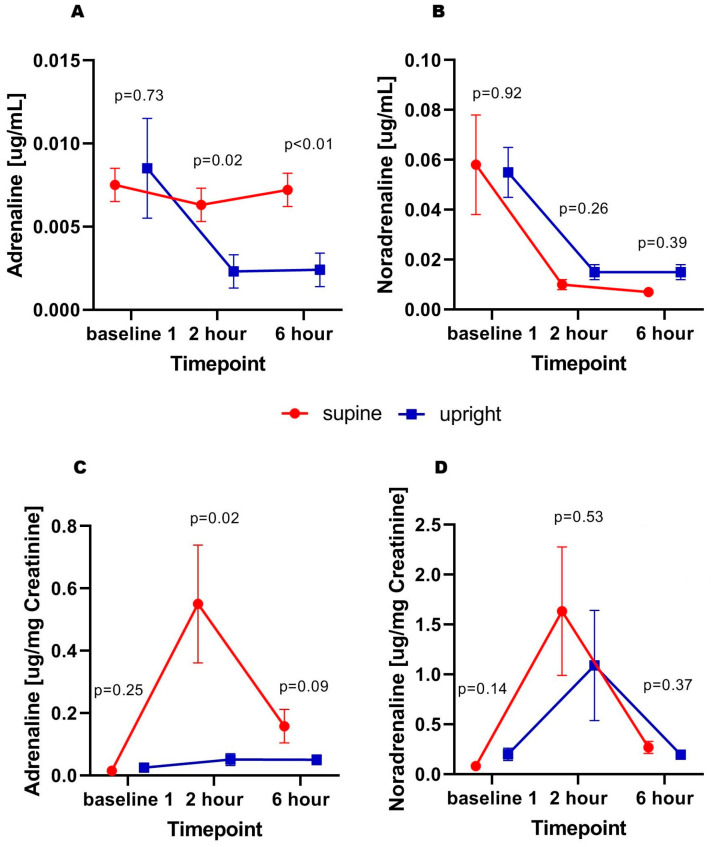
Adrenaline (**A**), noradrenaline (**B**) concentration; adrenaline (**C**) and noradrenaline concentration adjusted for urinary creatinine (**D**) in subsequent timepoints.

No statistically significant differences in aldosterone concentrations were found at subsequent time points. A similar pattern was observed for renin; however, despite the lack of statistical significance, there was a trend toward higher values in patients who remained in the upright position (Figure 3A,B). Regarding markers of adrenergic activity, higher urinary epinephrine concentrations were observed at 2nd hour (0.006 ± 0.001 vs. 0.002 ± 0.001 µg/mL; *p* = 0.02) and 6th hour (0.007 ± 0.001 vs. 0.002 ± 0.001 mL/h; *p* < 0.01) in supine patients compared to those in the upright position, while baseline concentrations were similar. Furthermore, this difference was preserved after adjustment for urinary creatinine concentration as a marker of renal filtration capacity. No significant differences were found with respect to norepinephrine—a similar pattern of changes was observed in both groups (Figure 4A–D).

## 4. Discussion

The presented study demonstrates several findings regarding the pathophysiology of the diuretic response in heart failure. Given the nature of the data obtained, as described in detail in the limitations discussion below, the study primarily reflects a descriptive tendency toward changes observed in patients rather than establishing direct causality:

1. No significant differences were found between the groups in terms of total urine output. Interestingly, those in the supine position presented a (non-significant) tendency to a more dynamic diuretic response.
2. Urinary sodium concentration, total and fractional sodium excretion were comparable between the groups at all (pre- and post-diuretic) timepoints. However, total sodium excretion in supine patients before furosemide administration showed a tendency (non-significant) for being higher.
3. Patients in both groups had similar aldosterone concentrations before and after diuretic administration. Likewise, no significant differences in renin secretion were observed; however, upright patients showed a trend toward higher renin levels than those in the supine position.
4. Interestingly, patients who remained in the supine position exhibited higher urinary adrenaline concentrations after diuretic administration. This effect was particularly evident after adjustment for urinary creatinine, reflecting glomerular filtration function.

In studies of a similar design conducted before the era of neurohormonal blockade, in different patient populations (e.g., with nephrotic syndrome or severe hypoalbuminemia), a significantly higher urine output and urinary sodium excretion were observed both at baseline and after diuretic administration in patients allocated to supine position group. Additionally, the fractional sodium excretion was higher between baseline measurements in the supine versus upright positions, with no post-diuretic differences [17].

A comparable effect—greater diuresis and sodium excretion (associated with higher eGFR)—was observed in a randomized trial administering 1 mg bumetanide to patients with liver cirrhosis or heart failure (analyzed as a single study group). Furthermore, plasma concentrations of noradrenaline, renin, and aldosterone tended to be higher in patients who remained upright during diuretic therapy [18].

Importantly, in heart failure patients treated with enalapril 10 mg twice daily, no significant differences in urine output or sodium excretion were observed between the supine and upright positions, whereas this effect was observed in patients receiving furosemide alone. Moreover, ACEI-treated patients exhibited higher plasma renin activity with a relatively large standard deviation across timepoints, while angiotensin II concentrations were significantly lower, confirming effective pharmacological blockade [19,20].

The changes observed between these populations following the introduction of ACEI therapy, supported by the findings of our study, suggest that the altered diuretic response profile between upright and supine patients may be a consequence of neurohormonal blockade. Its influence on renal function—most likely by (1) modulating vascular resistance—appears to diminish the role of pressure gradients in driving urine production under the strong stimulus of loop diuretic administration, and (2) in sodium avidity regulation during decongestion.

Despite a lack of clinically significant effect (no difference in urine output, urine dilution and natriuresis), the results have shown potential different mechanisms of this condition. On the one hand, high renin excretion in those being in the upright position (with potentially higher venous pressure at the kidneys’ level), and on the other hand, adrenergic drive in patients being supine, with potentially better right ventricle preload, and diminished hydrostatic pressure impact on venous pressure gradients.

Another issue should be noted: patients who remained in the supine position showed a more dynamic trajectory of urine excretion, which, in conjunction with the trend toward higher renin concentrations, may have contributed to the observed tendency toward urine concentration relative to baseline, as compared with upright patients (who exhibited a more “flat” diuretic response pattern). It was also reflected in (non-significant) higher urine dilution despite higher tendency for baseline urinary creatinine concentration. Nonetheless, the lack of statistical significance in these parameters potentially could be diluted due to the limited study population (which was discussed below).

Additionally, although the difference did not reach statistical significance, total sodium excretion tended to be higher before loop diuretic administration in patients assigned to the supine group, but spot urine sodium concentration remained highly similar. Notably, at baseline 1, supine patients also showed a tendency toward higher FeNa, with a diminished difference at baseline 2. Because all participants were initially in the supine position, this particular finding is more likely attributable to better renal function —reflected by higher serum creatinine and urea—in the supine in relation to the upright.

The study was subject to several limitations. First, the study group was small, resulting in limited statistical power. Therefore, the results should be interpreted with caution and considered exploratory and hypothesis-generating rather than definitive. Consequently, it increases the likelihood that meaningful associations may not have been detected, resulting in type 2 error. The extension of the study population was not possible due to the study’s financial limitations. Second, although the study was randomized, it was not blinded to the investigator, which may have introduced a certain degree of assessment bias. Third, the patient cohort was highly selected (with strict inclusion/exclusion criteria), which limits the generalizability of the findings to the broader population of patients with HFrEF; however, its role was to mechanistically show the pathophysiological patterns of diuretic response in relation to patients’ body position. Fourth, despite the lack of significant differences in urine output or natriuresis between groups adjusted for eGFR, the worse renal in upright patients could potentially confound the interpretation of the results. Fifth, total body water (including intravascular volume) was not objectively assessed prior to the study. Volume status and congestion were determined by the physician based on physical examination. Finally, the use of covariate or regression analyses was severely limited, preventing the exclusion of potential confounders (e.g., age, sex, kidneys’ function parameters) when evaluating diuretic response.

## 5. Conclusions

No clinically meaningful differences were observed between supine and upright AHF patients receiving neurohormonal blockade, although trends in renin and urinary adrenaline were noted. Further adequately powered studies are needed to confirm these findings.

## Figures and Tables

**Table 1:** Characteristics of the general population and comparison of the supine and upright groups.

Parameter	General Population	Supine	Upright	*p*-Value
Sex, men N (%)	10 (83%)	5 (83%)	5 (83%)	1.00
Age, years	74 ± 4	72 ± 8	76 ± 2	0.57
**Concomitant diseases**				
Hypertension, N (%)	10 (83%)	5 (83%)	5 (83%)	1.00
Diabetes mellitus, N (%)	7 (58%)	3 (50%)	4 (67%)	0.56
Chronic kidneys disease, N (%)	7 (58%)	2 (33%)	5 (83%)	0.08
COPD/Asthma, N (%)	2 (17%)	1 (17%)	2 (17%)	1.00
Myocardial infarction in past, N (%)	6 (50%)	2 (33%)	4 (67%)	0.35
**Echocardiography**				
LVDD, cm	65 ± 3	69 ± 3	62 ± 3	0.16
LVEF, %	28 ± 2	28 ± 3	28 ± 2	0.83
TAPSE, cm	14 ± 1	15 ± 1	12 ± 1	0.11
S’ TV, cm/s	7.0 ± 0.9	8.4 ± 1.5	5.9 ± 0.8	0.21
E/E’	26.5 ± 5.1	32.6 ± 13.1	22.8 ± 3.4	0.53
**Serum biochemical assessment**				
Leukocytes, 10^3^/µL	7.16 ± 0.51	6.61 ± 0.74	7.72 ± 0.68	0.29
Hemoglobin, g/dL	11.5 ± 0.5	10.8 ± 0.6	12.2 ± 0.8	0.18
Hematocrit, %	36 ± 2	34 ± 2	38 ± 2	0.27
Platelet count, 10^3^/µL	194 ± 17	187 ± 17	201 ± 32	0.72
Bilirubin, mg/dL	1.4 ± 0.2	1.5 ± 0.2	1.4 ± 0.3	0.71
AST, IU/L	39 ± 12	29 ± 4	49 ± 24	1.00
ALT, IU/L	25 ± 5	21 ± 1	30 ± 10	0.33
GGTP, IU/L	82 ± 20	58 ± 20	99 ± 29	0.46
Urea, mg/dL	70 ± 14	40 ± 7	100 ± 21	0.03
Creatinine, mg/dL	1.4 ± 0.2	1.0 ± 0.1	1.7 ± 0.3	0.03
Sodium, mmol/L	138 ± 1	139 ± 1	138 ± 1	0.72
Potassium, mmol/L	4.0 ± 0.1	3.9 ± 0.1	4.1 ± 0.2	0.49
Chloride, mmol/L	103 ± 2	101 ± 2	105 ± 2	0.18
NT-proBNP, pg/mL	15,162 ± 4490	9015 ± 2946	21,309 ± 8056	0.39
Aldosterone, ng/dL	18.1 ± 4.5	19.1 ± 8.6	17.2 ± 4.2	0.69
Renin, µIu/mL	123.3 ± 56.4	74.6 ± 66.9	172.1 ±93.1	0.42
**Baseline urine biochemical assessment**				
Urea, mg/dL	1096 ± 152	1050 ± 264	1142 ± 178	0.94
Creatinine, mg/dL	63.7 ± 13.1	70.9 ± 25.2	56.5 ± 9.7	0.70
Sodium, mmol/L	74.6 ± 10.2	74.5 ± 15.7	74.7 ± 15	0.99
Potassium, mmol/L	38.3 ± 4.6	44.3 ± 8.5	32.2 ± 2.8	0.23
Chloride, mmol/L	73 ± 11	70 ± 19	76 ± 14	0.81
Adrenaline, µg/mL	0.008 ± 0.001	0.008 ± 0.001	0.009 ± 0.003	0.73
Noradrenaline, µg/mL	0.056 ± 0.013	0.058 ± 0.023	0.055 ± 0.014	0.92

## Data Availability

The raw datasets generated during this study may be obtained from the corresponding author upon request under restrictions related to patient confidentiality, ethical approval, and data protection regulations.
